# Optimising Urinary Tract CT Scans for Renal Colic: Enhancing Efficiency and Reducing Radiation

**DOI:** 10.7759/cureus.97305

**Published:** 2025-11-20

**Authors:** Suleyman Ullah, Zayd M Noofel, Abbas K Khoja, Ala Bashir, Haris Shoaib, Sulaiman Hussain, Haider Chaudhary

**Affiliations:** 1 Department of Radiology, Basildon and Thurrock University Hospital, Basildon, GBR; 2 Department of Internal Medicine, Chelsea and Westminster NHS Trust, London, GBR; 3 Department of Urology, Nottingham University Hospitals NHS Trust, Nottingham, GBR; 4 Department of General Surgery, Worcestershire Acute Hospitals NHS Trust, Worcestershire, GBR; 5 Department of Trauma and Orthopaedics, Royal Bolton Hospital, Bolton, GBR; 6 Department of Trauma and Orthopaedics, Stoke Mandeville Hospital, Aylesbury, GBR; 7 Department of Oral and Maxillofacial Surgery, Cumberland Infirmary, Carlisle, GBR

**Keywords:** audit, computed tomography, ct urinary tract, quality improvement, radiation, radiology, renal colic

## Abstract

Background: In a locally performed audit, we found that the majority of computed tomography (CT) scans of the urinary tract performed for suspected renal colic did not meet the overscan criteria in line with national guidelines. We aimed to reduce unnecessary radiation in a local radiology department by designing a suspected renal colic radiology pathway.

Methods: We designed a retrospective, observational, single-centre study performed at Basildon & Thurrock University Hospital (BTUH), Basildon, United Kingdom. Using the department’s Picture Archiving and Communication System (PACS), this study reviewed 101 chronological CT scans of the urinary tract in two separate audit cycles between January and August 2025 that had been requested to investigate suspected renal colic, assessing whether they met criteria for overscan as per national standards. Our interventions included a vetting note offering a recommendation for the upper border of the scan (T11 vertebra), included for all CT urinary tracts requested for suspected renal colic. We also designed a poster for radiographers, highlighting the expected borders of the CT scan. The data were anonymised, and demographical information was collected. We calculated the overscan percentage by recording the number of total and overscan axial slices. Dose length product (DLP) data was obtained to quantify excess radiation exposure.

Results: In our initial audit cycle, we found that only 36% (18/50) of CT scans reviewed met criteria for overscan. Mean DLP overscan was calculated at 28.21 mGy·cm with extrapolated overscan radiation dosage measured at 0.423 mSv. Following the interventions, a further 51 scans were audited, revealing improved results; 70.6% (36/51) of CT scans reviewed met criteria for <10% overscan. Mean DLP overscan improved to 16.21 mGy·cm, and extrapolated overscan radiation dosage markedly improved to 0.243 mSv. Only 28% (14/50) of scans had correct positioning at the T11 vertebra level pre-intervention, improving to 56.9% (29/51) post-intervention. Compliance with the accepted standard increased by 96.1%. Average overscan decreased by 29.2%, and average excess radiation exposure decreased by 42.6%.

Conclusion: Our interventions resulted in adherence to the accepted standard doubling over a four-month period. Average ionising radiation exposure has been significantly reduced, which reduces the risk of carcinogenesis. Both interventions carried out in this study are low fidelity and are readily reproducible on a national scale. This study encourages radiology departments to audit radiation exposure from CT scans of the urinary tract for suspected renal colic, according to national standards. Future work should focus on multi-centre implementation to validate generalisability, assess long-term sustainability of behaviour change, and quantify the cumulative dose savings at a population level.

## Introduction

Computed tomography (CT) of the urinary tract, otherwise known as CT of the kidneys, ureters, and bladder (CT KUB), has emerged as the gold standard imaging modality for suspected renal colic, offering rapid acquisition and superior diagnostic performance in the detection of urolithiasis [1]. With a reported sensitivity of 95%-96% and specificity of 98% for urinary tract calculi, non-contrast CT of the urinary tract has largely supplanted plain abdominal radiography in contemporary clinical practice, demonstrating particular utility in identifying small, radiolucent, or anatomically complex stones [2,3]. Current guidelines from the British Association of Urological Surgeons (BAUS) recommend CT urinary tract imaging as the first-line investigation for adults presenting with acute flank pain suggestive of ureteric colic, given its robust diagnostic accuracy and capacity to identify alternative pathologies [4].

Despite its diagnostic superiority, optimisation of urinary tract CT protocols to balance diagnostic efficacy with radiation safety remains a critical challenge in contemporary radiological practice. This concern is particularly pertinent in patients with recurrent urolithiasis, a condition affecting approximately 50% of stone formers within five to 10 years of initial presentation. This patient cohort undergoes repeated imaging studies, accruing substantial cumulative radiation exposure over time [5]. The effective radiation dose from a standard urinary tract CT protocol is approximately 10 mSv per scan, equivalent to three years of natural background radiation and representing 50% of the U.K.'s annual occupational exposure limit of 20 mSv [6,7]. While Karim et al. (2017) estimated an attributable cancer risk of 35 per 100,000 CT urinary tract examinations, the clinical significance of this risk amplifies considerably with serial imaging, underscoring the importance of judicious protocol design and adherence to dose optimisation principles [6].

National imaging standards, established by the Royal College of Radiologists (RCR), stipulate that scan coverage extending beyond the upper pole of the highest kidney should not exceed 10% of total scan length [8]. This recommendation is predicated on evidence demonstrating that calculi above this anatomical landmark are exceedingly rare in the clinical context of suspected renal colic and that extended cranial coverage does not enhance diagnostic yield. Conversely, excessive superior scanning contributes to unnecessary radiation exposure without corresponding clinical benefit, prolongs examination time, and increases dose length product (DLP), practices fundamentally inconsistent with the As Low As Reasonably Achievable (ALARA) principles that govern all medical imaging practice [9]. These are fundamental principles of radiation safety that include considerations such as shielding, distance from the scanner, and exposure time [9].

Beyond radiation safety considerations, protocol non-compliance carries significant economic implications. Extending scan coverage beyond clinically indicated boundaries not only escalates patient radiation burden but also diminishes scanner efficiency, reducing patient throughput and increasing per-examination operational costs. Each additional centimetre of unnecessary scan length incrementally increases DLP and marginally extends staff time, energy consumption, and equipment utilisation. Evidence suggests that limiting scan coverage in accordance with the RCR guidance can reduce unnecessary resource expenditure and imaging costs and enhance departmental workflow efficiency without compromising diagnostic yield [10]. Thus, optimisation of urinary tract CT protocols offers dual benefits: enhanced patient safety through dose reduction and improved operational efficiency through resource stewardship.

While scan range reduction and dose optimisation are essential for patient safety, these measures must not compromise diagnostic confidence or image quality. Excessive dose reduction, irrespective of methodology, risks degrading spatial and contrast resolution, potentially hindering calculus detection and thereby negating the fundamental purpose of the examination. Accordingly, this audit was designed to evaluate institutional adherence to RCR and BAUS standards regarding urinary tract CT scan coverage, specifically, the use of a marginal excess beyond the upper pole of the highest kidney as the upper scan limit, and to assess the impact of protocol compliance on diagnostic accuracy, operational efficiency, and concordance with ALARA principles.

## Materials and methods

We designed a multi-cycle, retrospective, single-centre audit at Basildon & Thurrock University Hospital (BTUH), Basildon, UK, conducted against RCR iRefer and BAUS guidance for imaging in suspected renal colic. We reviewed 101 urinary tract CT scans, requested for suspected renal colic, in chronological order in two separate audit cycles between January and August 2025, which consisted of 50 pre-intervention scans and 51 post-intervention scans. 

All records were fully anonymised before analysis; no patient-identifiable data were retained. Appropriateness and scan extent were assessed against RCR/BAUS recommendations for CT scans of the urinary tract in acute renal colic (non-contrast CT as first-line in non-pregnant adults, performed promptly, and collimated to include the kidneys fully while minimising dose). 

Inclusion criteria involved consecutive referrals that met strict requirements. A urinary tract CT (non-contrast CT KUB) was performed with the patient having been referred from the emergency department. The indication for the scan had to be explicitly documented as renal colic, ureteric stone, nephrolithiasis, or any other equivalent description. Finally, a complete PACS record (images and final report) needed to be accessible for the data collection process.

Exclusion criteria included any scans or referrals involving pregnant women or patients under the age of 18 due to the radiation risk. Individuals with a solitary kidney (including a horseshoe kidney) or a transplant kidney were also excluded from the audit. Any patients with repeat imaging for the same indication within the prior three months were not included. Cases with cancelled or aborted studies or incomplete metadata precluding analysis were excluded from the data collection process.

For each audit cycle, we queried the Picture Archiving and Communication System (PACS) for the preceding 14 days of urinary tract CT requests, filtered by the above criteria. This yielded 50 patients in cycle one (1-14 January 2025) and 51 patients in cycle two (18 July-1 August 2025). Dataset fields included request text, modality, series parameters, timestamps, report, and dose metrics (DLP). 

All studies were reviewed in axial, coronal, and sagittal planes. Open coronal and axial series were reviewed side-by-side to identify the upper pole of the highest kidney on the coronal series. On the axial series, we counted slices from the most superior point of the highest kidney to the upper scan border in addition to noting the total axial slices. Following this, we calculated the overscan proportion (slices above the upper kidney pole / total axial slices). We then recorded the upper scan border vertebral level to support the protocol setting. T12 was identified on coronal images as the lowest vertebra with rib articulation; for confirmation, the sagittal plane was used to localise S1, which has a distinct morphology, and subsequently counted cranially. For each exam, we computed overscan DLP (total DLP × overscan proportion). We were then able to estimate the overscan effective dose (ED) utilising an abdomen-pelvis coefficient (k) of 0.015 mSv·mGy⁻¹·cm⁻¹ (Overscan ED = k x overscan DLP). This allowed translation of excess exposure into intuitive equivalents, e.g., background radiation or chest radiograph doses.

Findings from the initial audit cycle were presented at the Radiology Audit Meeting at BTUH on 21^st^ May 2025 (multidisciplinary attendance of consultants, registrars, radiographers, and PACS managers). The session covered national standards for CT scans of the urinary tract in renal colic, local quantitative results, and variance from standards, in addition to proposed intervention ideas with multidisciplinary team (MDT) feedback for implementation planning.

**Figure 1:**
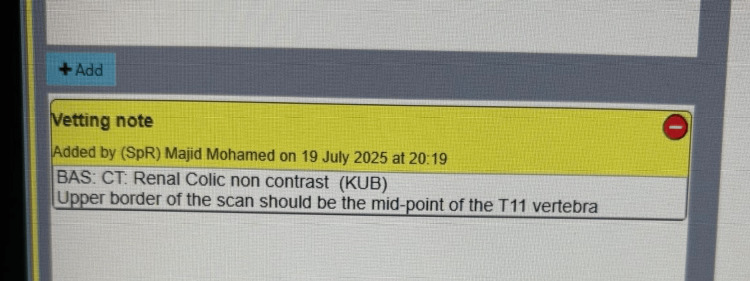
A new vetting note being utilised by a radiology registrar

**Figure 2:**
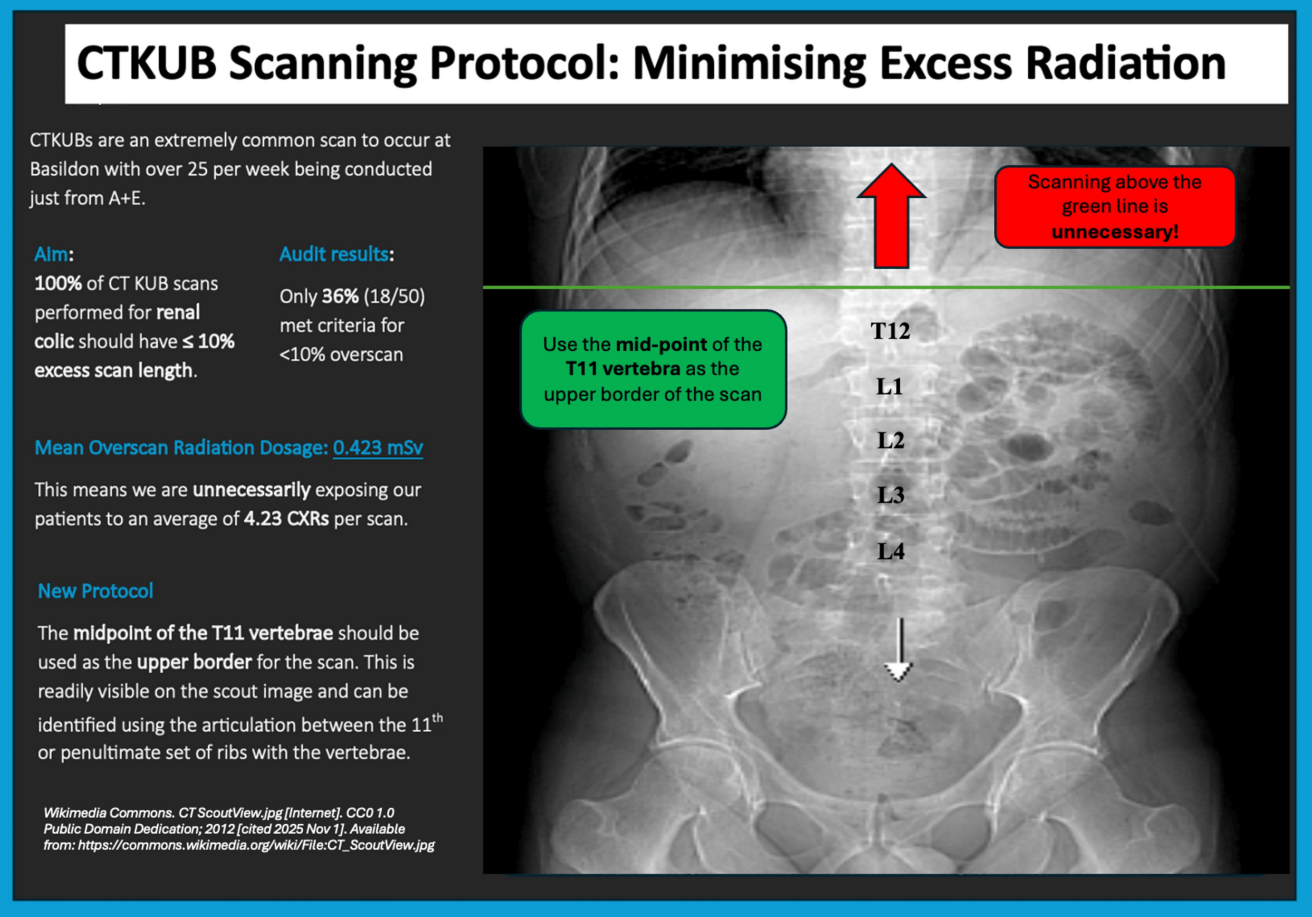
Poster demonstrating first-cycle audit findings and instructions for the new protocol, which was displayed in each CT control room within the hospital.

In order to improve compliance with national standards, we designed multiple interventions. The first aimed to optimise the current vetting process. The default vetting note for this indication was amended from “BAS: CT: Renal Colic non contrast (KUB)” to “BAS: CT: Renal Colic non contrast (KUB). The upper border of the scan should be the midpoint of the T11 vertebra.” The phrase now auto-populates when “renal colic” is entered (Figure 1). We facilitated targeted education for the radiographers, which included a brief teaching and a one-page crib sheet on scan coverage and vertebral landmarks. Lastly, we provided a visual aid in the CT control rooms in the form of a colourful, informative poster. This displayed an annotated scout image demonstrating T11 localisation and the rationale for limiting cranial coverage (Figure 2). Intervention content was aligned to RCR AuditLive recommendations on minimising urinary tract CT dose through appropriate collimation (upper pole included; avoid unnecessary cranial coverage).

Following the implementation of our interventions, we performed a second audit cycle, repeating the same case definition, extraction, and assessment to permit like-for-like comparison. Categorical outcomes (e.g., proportion with overscan >10%) have been summarised as counts and percentages with 95% CIs (Clopper-Pearson). Pre- and post-intervention comparisons of proportions have been calculated using chi-squared tests and Fisher’s exact test when expected counts were less than 5. Continuous or skewed measures (e.g., overscan proportion, overscan DLP, ED_overscan) have been compared using Mann-Whitney U tests, reporting medians and interquartile ranges, and Spearman’s Rho tests.

## Results

Data were collected from 50 patients in audit cycle one and 51 patients in audit cycle two (n = 101). Mean total axial slices were 642.0 ± 54.7 in cycle one and 694.2 ± 223.8 in cycle two (Table 1). Mean overscan (slices above the upper pole) was 79.0 ± 42.4 slices in cycle one and 58.0 ± 29.6 slices in cycle two, corresponding to mean overscan percentages of 12.1% ± 5.8% and 8.6% ± 4.4%, respectively.

**Table 1:** Summary of collected data for each audit cycle Statistical comparisons between audit cycles were performed using the chi-square test for categorical variables (χ² = 12.04, p = 0.00049) and–Mann-Whitney U test for continuous variables (U = 1716.0, p = 0.0028). DLP: dose length product

Cycle	N	Mean total axial slices	Mean overscan axial slices	Mean overscan percentage	Mean total DLP (mGy·cm)	Mean excess DLP (mGy·cm)	Number of patients exceeding 10% overscan	Percentage of patients exceeding 10% overscan (%)
Cycle 1	50	642.0 ± 54.7	79.0 ± 42.4	12.1 ± 5.8	219.9 ± 156.1	28.21 ± 31.63	32	64
Cycle 2	51	694.2 ± 223.8	58.0 ± 29.6	8.6 ± 4.4	193.1 ± 73.0	16.21 ± 11.06	15	29.4

Thirty-two of 50 (64.0%) patients in cycle one and 15 of 51 (29.4%) patients in cycle two exceeded the standard. This difference was statistically significant (χ² = 12.04, p = 0.00049; Fisher’s exact p = 0.00067). The relative risk of exceeding the standard in cycle two compared with cycle one was 0.46 (95% CI 0.29-0.74), indicating a statistically significant increase in the proportion of scans meeting the guideline standards in cycle two. 

Overscan percentage distributions were analysed using a non-parametric comparison (Mann-Whitney U), which found a statistically significant reduction in overscan percentage between both cycles (Mann-Whitney U = 1716.0, p = 0.0028).

DLP analysis used the recorded total DLP per patient (DLP in mGy·cm). Mean total DLP was 219.9 ± 156.1 mGy·cm in cycle one and 193.1 ± 73.0 mGy·cm in cycle two. Estimated excess DLP attributable to overscan (proportional allocation of total DLP to overscan slices) averaged 28.21 ± 31.63 mGy·cm in cycle one and 16.21 ± 11.06 mGy·cm in cycle two. There was no evidence of a statistically significant correlation between overscan percentage and total DLP (Spearman’s rho = 0.06, p = 0.53), indicating that higher overscan percentage did not strongly predict higher total DLP in this dataset.

**Figure 3:**
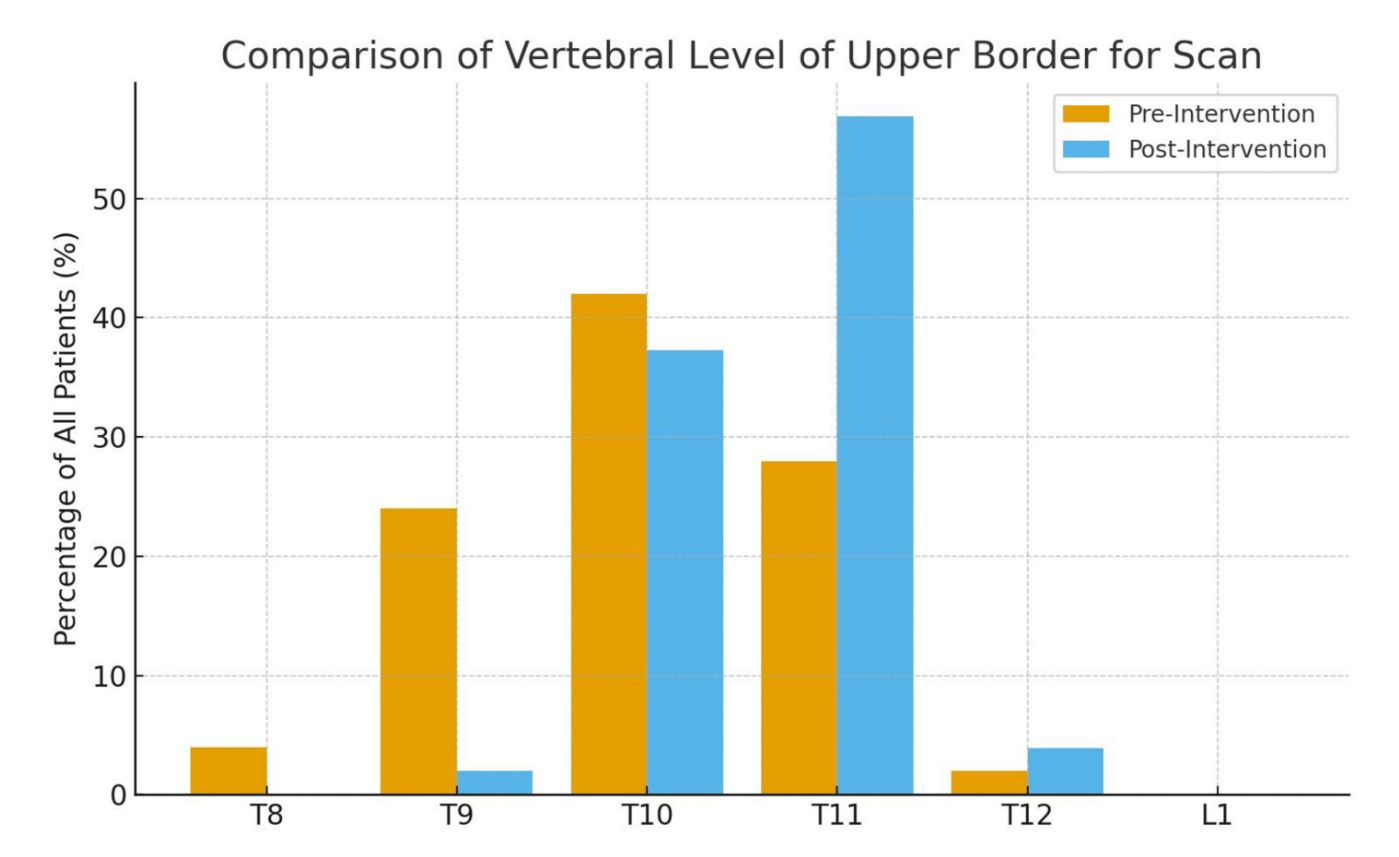
A grouped bar chart to compare the distribution of vertebral levels of the upper border for scans before and after the intervention. Cycle 1: 14/50 (28.0%) at T11; Cycle 2: 29/51 (56.9%) at T11. Statistical comparison performed using the chi-square test (χ² = 10.39, p = 0.0013).

In Figure 3, the results clearly show a shift towards correct positioning at T11, increasing from 28% (14/50) pre-intervention to 56.9% (29/51) post-intervention. In contrast, the proportion of scans taken at higher levels, such as T8, T9, and T10, decreased significantly. Before the intervention, 70% (35/50) of scans began at T10 or above. After the intervention, this proportion decreased to 39.3% (20/51), with most scans instead beginning at T11 (56.9% (29/51)). This indicates an overall improvement in the consistency and accuracy of scan positioning following the intervention.

**Figure 4:**
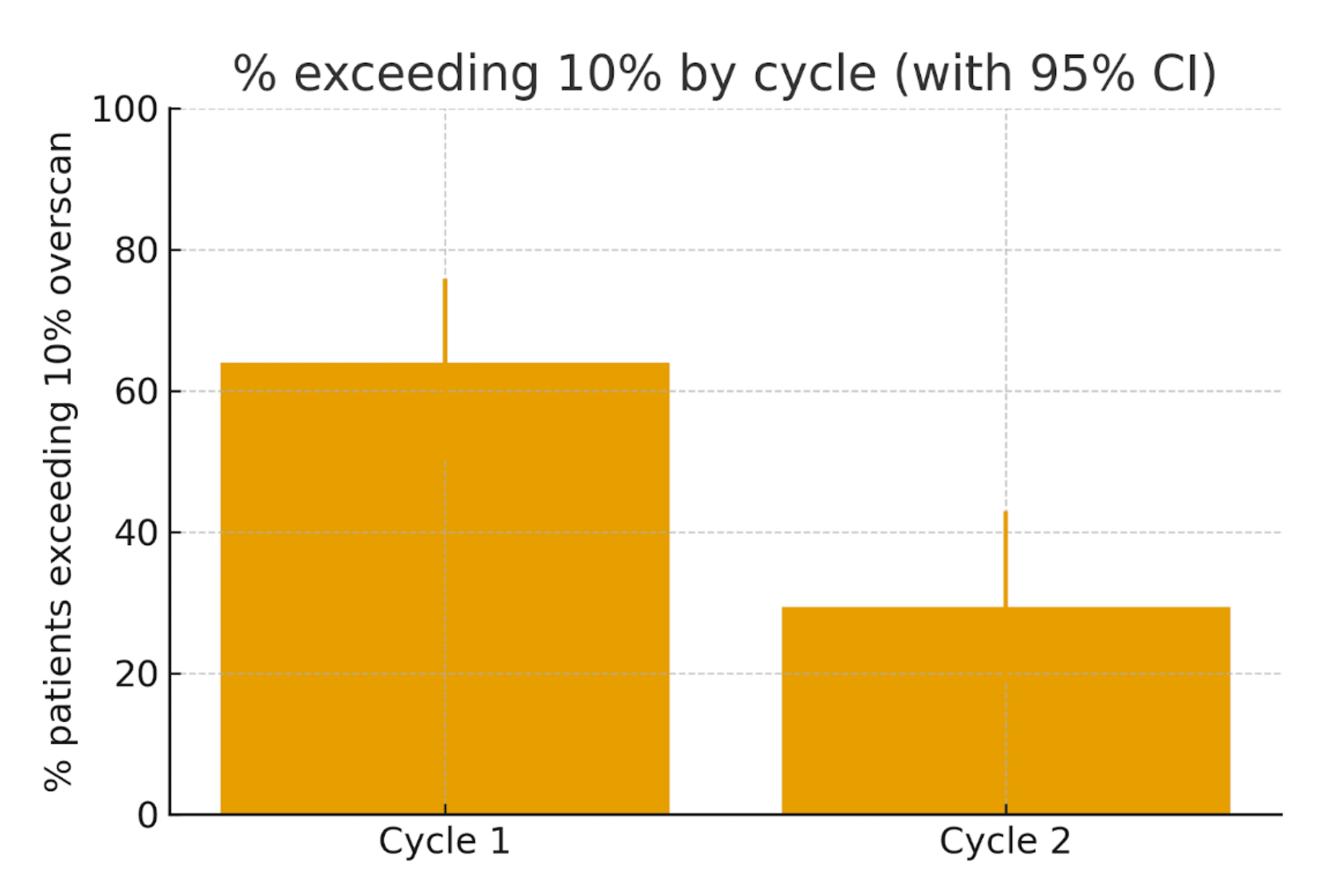
Percentage of patients exceeding the 10% overscan by cycle with 95% CIs Cycle 1: 32/50 (64.0%); Cycle 2: 15/51 (29.4%). Comparison between cycles performed using the chi-square test (χ² = 12.04, p = 0.00049). Error bars represent 95% CIs.

**Figure 5:**
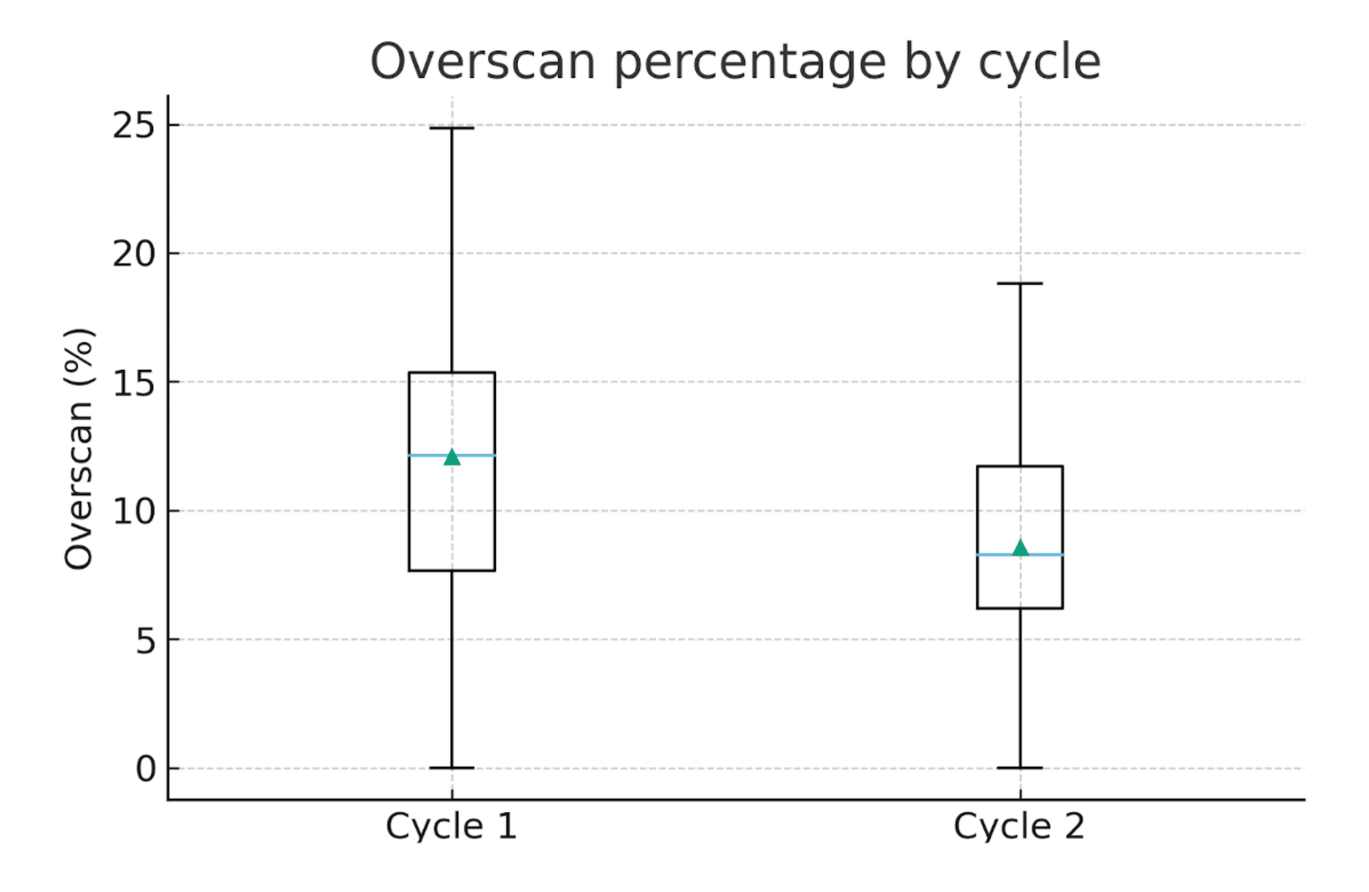
A boxplot comparing the overscan percentage between audit cycles. Statistical analysis was performed using the Mann–Whitney U test (U = 1716.0, p = 0.0028).

We demonstrated a reduction in over-scanning from 70% (35/50) in cycle one to 39.2% (20/51) in cycle two. Statistical analysis using the chi-squared test showed this reduction to be of significance (χ² = 10.39, p = 0.0013), which indicates a marked improvement rather than random variation. Overall, cycle two had significantly fewer scans exceeding the 10% overscan standard and a lower estimated excess DLP compared with cycle one (Figures 4, 5).

## Discussion

This multi-cycle audit demonstrates that targeted, pragmatic interventions in departmental workflow can substantially improve adherence to national scan-range standards for urinary tract CT imaging. The implementation of a pre-populated vetting template specifying cranial scan limits, coupled with dedicated educational sessions for radiography staff and strategically positioned visual aids within CT control rooms, reduced the proportion of scans exceeding the 10% overscan threshold (standard set by the RCR [11]) from 64.0% (32/50) in cycle one to 29.4% (15/51) in cycle two, a statistically significant 34.6% reduction (χ² = 12.04, p = 0.00049). Mean overscan percentage decreased significantly from 12.1% ± 5.8% to 8.6% ± 4.4% (Mann-Whitney U = 1716.0, p = 0.0028), while estimated excess DLP attributable to overscan was nearly halved from 28.21 ± 31.63 mGy·cm to 16.21 ± 11.06 mGy·cm. Critically, these improvements were achieved without compromising diagnostic adequacy, as no underscanning occurred in either audit cycle. These findings underscore the feasibility of achieving meaningful protocol compliance through multifaceted quality improvement strategies. 

Rationale for T11 midpoint as the upper border landmark

The selection of the T11 vertebra midpoint as our upper scan limit was evidence-based rather than arbitrary, drawing from established anatomical principles and prior optimisation studies. Uldin et al. (2020) identified T11 as a reliable anatomical landmark that is fixed in position relative to the kidneys, sufficiently proximal to reduce scan length, adequately distant to prevent underscanning, and readily identifiable on coronal scout images through rib articulation with thoracic vertebrae [12]. Their study demonstrated that implementing T11 as the superior border reduced mean overscan percentage from 28.2% to 10.6% with zero cases of underscanning, findings that closely parallel our own results.

Multiple subsequent studies have corroborated T11 as an effective reference point. Research has shown that T11 provides comprehensive visualisation of the kidneys while limiting unnecessary radiation to surrounding structures, with the landmark demonstrably reducing overscanning without compromising diagnostic utility [13]. 

Recent multicentre data confirm that 97.6% of scans fully visualise both kidneys when commencing from T11, compared to only 79.2% when starting at T12, establishing T11 as a practical balance between safety margins and dose optimisation [5].

By specifying the midpoint of T11 rather than simply "T11," we provided radiographers with precise, actionable guidance that reduced interpretive variability and enhanced protocol adherence. This specificity was critical to achieving the significant reductions in overscan observed between audit cycles.

Comprehensive intervention strategy

The efficacy of our interventions can be attributed to their comprehensive and complementary nature. Educational engagement with radiography staff constituted our primary and arguably most impactful intervention, fostering awareness of appropriate technical parameters and cultivating a culture of protocol adherence. The effectiveness of targeted radiographer education in optimising CT acquisition parameters and reducing radiation dose is well-established in the literature [14,15].

Our pre-populated vetting template functioned as a form of clinical decision support system, ensuring that appropriate scan parameters remained consistently visible to operators during protocol selection. Such decision support tools have been shown to reduce cognitive burden in high-acuity clinical environments, thereby enhancing protocol compliance and patient safety [16,17]. Standardisation of scanning parameters through readily accessible, prepopulated templates at the point of protocol assignment predictably enhances adherence to established guidelines [18]. The visual reminder poster, positioned prominently within CT control rooms, served to reinforce educational messaging and extend its reach across staff members, including those unable to attend formal training sessions. Evidence supports the efficacy of strategically placed educational posters as sustainable behaviour modification tools when maintained in protected, high-visibility locations over extended periods [19]. 

Objective quantification and reproducibility

A notable strength of this audit lies in the objective quantifiability of outcomes through DLP measurement and derived ED calculations, as well as direct assessment of overscan percentage. This approach enabled robust, evidence-based evaluation of intervention effectiveness. Furthermore, the use of slice count as a primary metric offers portability across different scanner platforms, reconstruction software, and institutional settings. This methodological reproducibility facilitates wider implementation and comparative analysis across multiple centres, supporting potential regional or national scaling of these quality improvement strategies.

The 31% reduction in overall overscan prevalence between cycles (from 70% (35/50) to 39.2% (20/51), χ² = 10.39, p = 0.0013) represents not merely statistical significance but a clinically meaningful improvement in patient radiation safety. 

Moreover, the absence of correlation between overscan percentage and total DLP (Spearman's rho = 0.06, p = 0.53) suggests that overscan primarily reflects scan range rather than patient-specific factors such as body habitus or tissue density. This finding reinforces the potential for protocol-level interventions, rather than patient-level adjustments, to achieve substantial dose reductions across diverse populations.

Cost-effectiveness and resource implications

An essential yet often underappreciated aspect of quality improvement initiatives is their economic sustainability. The interventions implemented in this audit were deliberately designed to be cost-effective, requiring minimal financial outlay and limited personnel time investment. The automated vetting note modification involved a one-time text amendment in the radiology information system, implemented by existing IT support staff within minutes. Educational sessions were delivered during routine departmental audit meetings, utilising protected time already allocated for quality improvement activities, thereby incurring no additional salary costs. The visual poster was produced in-house, using freely available departmental resources and standard printing facilities, with a negligible material cost of approximately £5.

Importantly, these low-cost interventions did not require additional staffing, external consultancy, disruption of workflow, expensive software upgrades, or hardware modifications. The entire quality improvement cycle was conducted using existing infrastructure, demonstrating that meaningful radiation dose optimisation can be achieved without substantial capital investment. This contrasts sharply with alternative dose-reduction strategies, such as iterative reconstruction algorithms or advanced CT scanner technology, which may require significant financial commitment and prolonged procurement processes.

Furthermore, the operational benefits extend beyond direct cost savings. By reducing unnecessary scan coverage, we improved scanner efficiency and patient throughput. Each centimetre of excess scan length incrementally increases acquisition time, DLP, and examination duration. Eliminating this waste enhances departmental workflow, reduces patient waiting times, and optimises staff utilisation. Evidence suggests that limiting scan coverage in accordance with RCR guidance can reduce unnecessary resource expenditure, lower imaging costs, and enhance departmental workflow efficiency without compromising diagnostic yield [20]. 

From a healthcare system perspective, the scalability and reproducibility of these interventions represent substantial value. With renal colic affecting approximately 10% of the population and recurrent stone disease occurring in 50% of stone formers within five to 10 years, the cumulative radiation exposure across patient populations is considerable. Our interventions, if replicated across regional or national imaging networks, have the potential to deliver population-level dose reductions without requiring centralised funding or infrastructure investment. This positions our approach as not merely clinically effective but economically pragmatic, a critical consideration in resource-constrained healthcare environments.

Limitations and future directions

However, several limitations warrant acknowledgement. The estimation of ED using standardised conversion coefficients introduces potential inaccuracy, as true ED varies with tissue weighting factors, patient body habitus, and scanner specifications [21]. While our ED-based overscan calculations adequately identified institutional trends, measurement imprecision may have introduced undetected bias into our dataset. Additionally, this single-centre study encompassed a modest 101 examinations, limiting generalisability. The scalability of our interventions remains uncertain, particularly regarding the feasibility of delivering consistent educational interventions across larger radiography cohorts at regional or national levels. The brief interval between audit cycles introduces the possibility of Hawthorne effect bias, whereby staff adherence to protocols may have been artificially enhanced by awareness of ongoing performance evaluation [22]. Finally, the sustained effectiveness of educational posters is contingent upon optimal placement and long-term visibility, factors that may vary significantly across institutions and prove challenging to maintain [19]. 

Long-term sustainability of protocol improvements requires systematic reinforcement through regular, annual re-audit cycles and ongoing educational initiatives to ensure departmental awareness of evolving guidelines and technical standards. Our next planned intervention involves establishing a 'change champion' network, wherein selected radiography staff receive intensive training and subsequently deliver peer-led educational sessions within their teams. Evidence demonstrates that protocol modifications taught by local champions and reinforced through brief, workplace-based peer interactions achieve superior adherence rates and greater behavioural sustainability across clinical teams and institutional sites [23]. This approach will be strengthened through regular performance feedback, as iterative audit-feedback cycles have proven highly effective in achieving durable change in practice [24]. Using the data collected through this audit, provide a foundation for developing a sustainable quality improvement model suitable for implementation across partner institutions within our hospital trust through peer-to-peer educational frameworks. Such collaborative teaching models have demonstrated the capacity to propagate sustained practice change and establish best practices at scale, prior to broader regional or national dissemination [25].

## Conclusions

This multi-cycle audit demonstrates that targeted, multi-modal interventions can achieve significant improvement in adherence to national urinary tract CT imaging standards. By optimising scan collimation without compromising diagnostic accuracy, we simultaneously advanced ALARA principles and enhanced operational efficiency, objectives of paramount importance in the management of recurrent urolithiasis, where cumulative radiation exposure poses genuine long-term risk. The interventions described are scalable and readily adaptable to diverse institutional settings, requiring minimal resource investment yet yielding measurable benefits in radiation dose reduction and protocol standardisation. Future work should focus on multi-centre implementation to validate generalisability, assess long-term sustainability of behaviour change, and quantify the cumulative dose savings at a population level. Establishing such standardised protocols across regional imaging networks may represent a pragmatic pathway toward meaningful radiation dose optimisation in contemporary urological imaging practice.
